# Longitudinal Serial Mediation Study after the 2023 Earthquake in Türkiye: Associations Between Difficulties in Emotion Regulation, Psychological Distress, Resilience and Mental Well-Being

**DOI:** 10.1007/s11126-025-10130-0

**Published:** 2025-03-15

**Authors:** Sinan Okur, Seydi Ahmet Satıcı, Beste Erdinç, Yusuf Akyıl

**Affiliations:** 1 Department of Educational Sciences, National Defense University, Turkish Air Force Academy, 34149 Istanbul, Türkiye; 2 Faculty of Education, Department of Psychological Counseling, Yıldız Technical University, Istanbul, Türkiye; 3 Avcılar Anatolian High School, Ministry of National Education, Istanbul, Türkiye

**Keywords:** Difficulties in emotion regulation, Psychological distress, Resilience, Mental well-being, Longitudinal serial mediation study

## Abstract

Although the concepts of mental well-being, difficulties in emotion regulation, resilience, and psychological distress have been investigated in cross-sectional studies, their absence from a longitudinal study demonstrates a gap in the literature. Following the earthquake disaster in Türkiye, addressing these concepts together in a longitudinal study may offer important implications for the field of mental health. The longitudinal mediation of resilience and psychological distress in the relationship between difficulties in emotion regulation and mental well-being was investigated in a Turkish adult sample. The study sample consisted of 219 participants aged between 18 and 45 (51.1% male, *M*_*age*_ = 31.60, *SD* = 7.19). To avoid the limitations of cross-sectional studies, data for the current study were examined at three-month intervals and at two time points in a cross-lagged panel model with a half-longitudinal design to investigate the mediating role of resilience and psychological distress between difficulties in emotion regulation and mental well-being. The analysis found that resilience and psychological distress played a longitudinal mediating role in the relationship between emotion regulation challenges and mental well-being. In conclusion, people's ability to regulate their emotions, be resilient, and avoid psychological distress may improve their mental health. These findings underscore the importance of integrative interventions that simultaneously target difficulties in emotion regulation, resilience, and psychological distress to better support mental well-being in post-disaster contexts.

## Introduction

Individuals go through a lot of events in their lives, which don't stop. Although some of these events are difficult for people to deal with, the ongoing structure of life requires individuals to live their lives in a functional way. Resilience is defined as the ability to persevere in the face of difficult experiences and environmental risks, as well as to overcome adversity. A resilient person can adapt to adversity without developing psychological disorders like post-traumatic stress disorder [1, 2]. In other words, resilience strengthens people in the face of difficult circumstances. Psychological distress is a disturbing emotional state that an individual experiences in response to a specific stressor or need, resulting in temporary or permanent harm [3]. Studies have demonstrated a negative relationship between resilience and psychological distress [4]. Distress factors, also known as difficult life conditions, can allow people to live their lives as normally as possible, as long as they remain resilient. According to Pakalniškienė et al. [5], resilience levels are linked to whether individuals experience psychological distress during stressful situations or not.

Individuals may want to be resilient for a variety of reasons. Resilience is closely related to positive thoughts about oneself, the world, and the future. Resilient individuals tend to have more positive thoughts, greater life satisfaction, and lower depression levels [6]. Similarly, resilience is inversely related to negative mental health indicators and positively correlated with positive mental health indicators. In other words, people with more depression, anxiety, or negative influence have lower levels of resilience, whereas people with more life satisfaction or positive affect have higher levels of resilience [7]. This suggests that resilience is linked to both mental health and emotional regulation. Life satisfaction, subjective happiness, and psychological functioning all reflect people's mental health [8]. Research indicates that resilient people have high levels of well-being [9, 10].

Resilience is the risk that is often linked to bad or unwanted outcomes, as well as the protective factors that help people grow in the way they want to [11]. Emotion regulation skills, self-awareness, and the ability to envision the future are among the most important determinants or drivers of resilience, as is a motivational system that pushes the individual to learn, grow, and adapt to their environment [12]. These explanations highlight the importance of investigating the relationships between the concepts of resilience and emotion regulation.

Emotion regulation refers to the external and internal processes that monitor, evaluate, and modify emotional responses [13]. Emotion regulation involves various processes, including identifying and labeling emotions, accepting and tolerating them, enhancing positive emotions, reducing susceptibility to negative emotions, as well as utilizing relaxation techniques, breathing exercises, and distraction strategies [14]. Emotion regulation, like well-being, is positively associated with resilience [15, 16]. Research indicates that individuals who struggle with emotion regulation and dysregulation experience lower levels of well-being [17, 18]. A study found that high levels of well-being predicted low levels of difficulties in emotion regulation [19]. Furthermore, psychological distress, like emotion regulation, is associated with lower well-being [20]. According to research, using high levels of resilience and adaptive emotion regulation strategies reduces anxiety, depression, insomnia, and fears, resulting in improved well-being [21]. Another study that looked at these concepts together found that resilience and emotion regulation are protective factors that reduce the distress caused by a negative situation [22].

### The Present Study

The present study will use a longitudinal serial mediation analysis to test the mediating role of psychological distress and resilience in the relationship between difficulties in emotion regulation and mental well-being. Individuals are more likely to react in a specific way because of a felt emotion, even if they are not required to. This flexibility in how emotions are expressed allows people to regulate their feelings. As previously stated, well-being is linked to emotions, so individuals must regulate their emotions [23]. Furthermore, emotion regulation provides insight into the emotional component of mental functioning [24]. Therefore, the research suggests that difficulties in emotion regulation, psychological distress, and resilience could potentially influence mental health.

In their daily lives, people may encounter a wide range of emotions, as well as difficult experiences and distress. Research suggests that experiencing natural disasters, such as earthquakes, markedly elevates the likelihood of developing psychological disorders, including post-traumatic stress disorder (PTSD), depression, and anxiety [25]. Evidence indicates that in the aftermath of such events, individuals frequently endure intensified feelings of fear, helplessness, and distress, which may persist over time and adversely affect their mental well-being [26]. In 2023, two major earthquake disasters centered in the Kahramanmaraş province of Türkiye occurred. These earthquakes directly and indirectly affected thousands of people in Türkiye. Hundreds of thousands of people whose homes were damaged or completely destroyed faced serious physiological problems. In addition, after the earthquake in Türkiye, people have been experiencing various emotional difficulties and psychological problems. According to assessments carried out in the aftermath of the earthquake, a considerable proportion of the population displayed signs of psychological distress, such as a propensity to cry, angry outbursts, changes in appetite, and almost all reported altered sleep patterns [27]. These conditions make it difficult for individuals to be resilient and emotionally stable. The prevalence of PTSD among earthquake survivors further complicates their ability to maintain emotional equilibrium [28]. In addition, due to the situation experienced, individuals are unlikely to have excellent mental health. The loss of close family members and fear of aftershocks have been explained as significant risk factors contributing to the deterioration of mental health among survivors [29]. It may be difficult for individuals to maintain a positive outlook on their well-being due to difficulties with emotion regulation, distress, or lack of resilience. Studies have revealed that difficulties in emotion regulation contribute to the development and maintenance of PTSD following exposure to natural disasters [30]. Based on all these research findings, the current study was necessary because there were no studies in the literature that examined these concepts together. Although certain elements, including resilience and emotion regulation, have been examined separately, thorough studies examining how these elements interact in the context of psychological discomfort brought on by earthquakes are scarce. Furthermore, longitudinal serial analysis should be used to investigate the relationships between the related concepts in the current study in order to better understand the causal link between these variables. Longitudinal studies are essential to discern the temporal sequence and causal relationships among emotion regulation difficulties, resilience, and psychological outcomes in disaster-affected populations. The current research will help people take appropriate actions and better understand the factors that influence their mental health after an earthquake. In this context, the following hypotheses will be tested in this study:

- *H1.* Psychological distress will longitudinally mediate the relationship between difficulties in emotion regulation and mental well-being.
- *H2.* Resilience will longitudinally mediate the relationship between difficulties in emotion regulation and mental well-being.
- *H3.* Psychological distress and resilience will jointly have a longitudinal serial mediating effect on the relationship between difficulties in emotion regulation and mental well-being.

## Method

### Participants and Procedure

This longitudinal study was conducted at three-month intervals in the first half of 2024. The participant form was completed online. Before collecting research data, necessary permissions were obtained from the National Defense University Humanities and Social Sciences Ethics Committee (Reference Number = E-35592990-050.04-3790156). In addition, this study complied with the ethical standards set forth in the 1964 Helsinki Declaration and its subsequent updates. Professional ethical principles were adhered to throughout the research. Then, the research data was collected using a web-based form. The link to the online form was sent to the participants via the authors' social media accounts. Prior to participating in the study, each participant provided informed consent. Participants participated in the study voluntarily, and no fee was paid to the participants throughout the research. The online form was designed so that participants could withdraw at any time and resubmit once all questions had been answered.

The longitudinal data included 278 participants in time one and 282 participants in time two. The questionnaires for the matching study included the last three letters of the mother's name, the first three letters of the father's name, and a section with the pseudonym. The final sample included 219 adult participants who participated in both waves. Possible reasons for data loss across the two waves included participant dropout due to changes in contact information or personal circumstances, as well as incomplete or invalid questionnaires that could not be matched. The final sample included 107 females (48.9%) and 112 males (51.1%). Participants' ages ranged from 18 to 45 years (*M*_age_ = 31.60, *SD* = 7.19). The majority of the participants are high school graduates (*n* = 142, 64.8%).

### Measures

#### Warwick-Edinburgh Mental Well-Being Scale Short Form

Tennant et al. [31] developed a scale to assess well-being, one of the fundamental concepts of positive psychology, in an adult sample. Demirtaş and Baytemir [32] adapted this measurement tool to Turkish culture, introducing a valid and reliable measurement tool for adults to assess mental well-being in Turkish literature. The Cronbach's alpha reliability coefficient for the scale was calculated to be 0.84, and its reliability was understood. In addition, this scale has the desired fit values. The scale contains a total of seven items (e.g., “*I feel useful*”), each of which represents one dimension. The Likert scale has a response range of 1 (*never*) to 5 (*always*). The scale can produce a score as low as 7 and as high as 35. A high score on the scale indicates a high level of mental well-being. In this study, the Cronbach’s alpha value of this scale was 0.845 at Time 1 and 0.881 at Time 2.

### Difficulties in Emotion Regulation Scale-8

Developed the scale to assess the difficulties in emotion regulation in adults [17]. This scale was adapted into Turkish by Ekşi and Erik [33]. The scale is composed of eight items (e.g., “*When I feel distressed/bad, it takes me a long time to feel better*”) and four sub-dimensions: "*goal*", "*impulse*", "*non-acceptance*", and "*strategy*". The internal consistency value for the sub-dimensions of the scale was calculated to be between 0.68 and 0.77, while the internal consistency value for the overall scale was calculated to be 0.87. It was found that the scale had adequate fit indices. The scale's response options are of the five-point Likert type (*1* = *almost never; 5* = *almost all the time*). Depending on the answers given on this scale, scores between 8 and 40 can be obtained. Difficulties in emotion regulation are more likely to be indicated by higher scores. In the current study, the Cronbach’s alpha coefficient of this scale was calculated as 0.876 at Time 1 and 0.885 at Time 2.

### K-10 Psychological Distress Scale

This scale, developed by Kessler et al. [34], was adapted into Turkish by Altun et al. [35]. The scale's Cronbach alpha reliability value is 0.95. The Likert scale, which has one dimension and 10 items (e.g., “*How often have you felt worthless about yourself this month?*”), is a five-point scale with answer options ranging from 1 (*never*) to 5 (*always*). The range of points that can be obtained from the scale is from 10 to 50, and results that are higher indicate a greater degree of psychological distress. In this study, the Cronbach’s alpha of the scale was 0.930 at Time 1 and 0.935 at Time 2.

### Brief Psychological Resilience Scale

This scale, developed by Smith et al. [36], aims to determine the resilience level of individuals. Doğan [37] adapted the scale to Turkish culture. This scale contains six items (e.g., “*I get through difficult times with little distress*”). The Turkish version's internal consistency coefficient is 0.83. Additionally, the fit index values ​​of the scale are at a sufficient level. The Likert scale is a five-point Likert scale (*1* = *not at all appropriate; 5* = *completely appropriate*). The scale is capable of generating scores ranging from 6 to 30. Possible higher scores mean that individuals have higher resilience. In the current study, the Cronbach’s alpha reliability coefficient of the scale was found to be 0.866 at Time 1 and 0.852 at Time 2.

### Data Analysis

In this study, SPSS was used for descriptive statistics and correlation analysis, JASP for reliability analysis, and AMOS statistical program for structural equation modeling. First, descriptive statistics, reliability analysis, and correlation analysis of the concepts were conducted. Using structural equation modeling, we investigated whether psychological distress and resilience mediate the longitudinal serial relationship between difficulties in emotion regulation and mental well-being. Difficulties in emotion regulation are the model's independent variable. Psychological distress and resilience are serial mediators. Mental well-being is the dependent variable.

In the analyses of this study, parceling technique was used. Little et al. [38] emphasized that this technique should be preferred to reduce measurement errors when using one-factor scales. In addition, Nasser-Abu Alhija and Wisenbaker [39] stated that this method is useful in terms of ensuring measurement normality and reliability. Considering all these reasons, parceling method was used for psychological distress, resilience, and mental well-being variables in this study. Apart from this, the model's goodness of fit can be measured using various indices, such as χ^2^ to degrees of freedom, Comparative Fit Index (CFI), Tucker-Lewis Index (TLI), Normed Fit Index (NFI), Incremental Fit Index (IFI), Goodness of Fit Index (GFI), and Standardized Root Mean Square Residual (SRMR). Finally, the longitudinal mediation model was tested using 5,000 bootstrap samples. According to Kline [40], CFI, IFI, TLI, NFI, and GFI values of 0.90 or higher are generally considered adequate model fit, whereas values of 0.95 or higher are preferred for good model fit. Furthermore, Kline [40] suggests that an SRMR cut-off value of 0.08 or lower indicates a satisfactory model fit.

## Results

Table 1 displays information about the measures, including means, standard deviations, skewness, kurtosis, and reliability. Table 1 also shows that there are significant correlations between difficulties in emotion regulation, psychological distress, resilience, and mental well-being. Emotion regulation difficulties at T1-2 have a significant negative correlation with mental well-being and resilience at T1-2. At T1-2, however, it has a significant positive correlation with psychological distress. Psychological distress at T1-2 has a significant positive relationship with resilience and mental well-being. Furthermore, at T1-2, there are strong positive relationships between resilience and mental well-being.

**Table 1:** Descriptive statistics, normality findings, reliabilities and correlation results for the study variables

Variable	1	2	3	4	5	6	7	8
1. Difficulties in emotion regulation T1	–							
2. Difficulties in emotion regulation T2	.57**	–						
3. Psychological distress T1	.57**	.33**	–					
4. Psychological distress T2	.38**	.51**	.53**	–				
5. Resilience T1	-.61**	-.39**	-.45**	-.24**	–			
6. Resilience T2	-.44**	-.51**	-.23**	-.36**	.56**	–		
7. Mental well-being T1	-.49**	-.36**	-.60**	-.47**	.44**	.30**	–	
8. Mental well-being T2	.39**	-.50**	-.42**	-.70**	.31**	.51**	.63**	–
Mean	21.06	20.27	27.12	25.79	19.25	19.34	25.06	25.30
SD	6.50	6.82	9.13	9.58	4.64	4.46	4.76	5.10
Skewness	.119	.293	.246	.414	-.114	.154	-.490	-.519
Kurtosis	-.709	-.317	-.567	-.498	.366	.554	.285	.320
McDonald ω	.878	.888	.932	.936	.864	.853	.856	.887
Cronbach α	.876	.885	.930	.935	.866	.852	.845	.881
Guttman λ6	.878	.887	.942	.944	.869	.852	.843	.875

^**^p < .05

### Structural Equation Modeling

First, different measurement models were evaluated. The first wave measurement model was good-fitted to the data (χ^2^ = 65.832, *df* = 29, χ^2^/df = 2.27, CFI = 0.973, TLI = 0.958, NFI = 0.953, IFI = 0.973, GFI = 0.944, SRMR = 0.043) and the second wave (χ^2^ = 99.827, *df* = 29, χ^2^/df = 3.33, CFI = 0.953, TLI = 0.927, NFI = 0.935, IFI = 0.954, GFI = 0.921, SRMR = 0.050). After validating the measurement model, the longitudinal structural model was investigated.

**Fig. 1:**
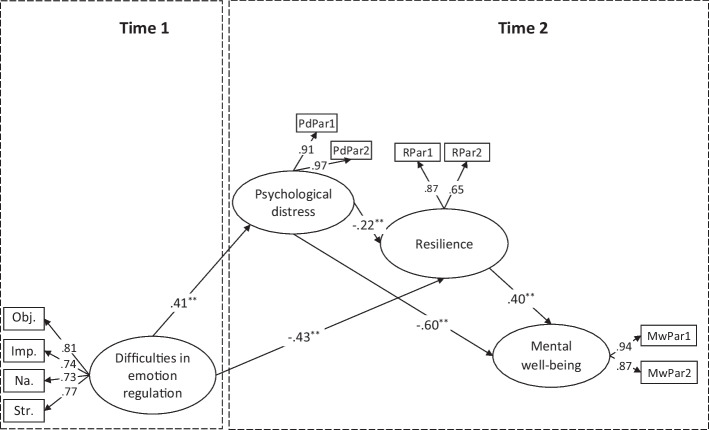
Structural equation modeling for the longitudinal serial mediation model. ^**^p < .01, Obj: Objective; Imp: Impulse; Na: Non-acceptance; Str: Strategy; PdPar: Parcel of psychological distress, RPar: Parcel of resilience, MwPar: Parcel of mental well-being

The longitudinal structural model is based on working hypotheses that investigate whether psychological distress and resilience play a role in longitudinal serial mediation of the effect of difficulties in emotion regulation on mental well-being while controlling for the role of baseline measurements. Figure 1 demonstrates that the longitudinal serial mediation model fit was acceptable (χ^2^ = 57.196, *df* = 30, χ^2^/df = 1.90, CFI = 0.980, TLI = 0.969, NFI = 0.959, IFI = 0.980, GFI = 0.952, RMSEA = 0.064, and SRMR = 0.046).

Difficulties in emotion regulation have a significant effect on psychological distress and resilience. Moreover, psychological distress and resilience have significant effects on mental well-being.

Next, bootstrapping was used to look into indirect effects (see Table 2). Significant indirect effects of T2 psychological distress (β = −0.325, bootstrapping CI = [−0.440, −0.210]) and T2 resilience (β = −0.394, bootstrapping CI = [−0.525, −0.258]) suggest that both variables may separately mediate the relationship between T1 difficulties in emotion regulation and T2 mental well-being. The relationship between T1 difficulties in emotion regulation, T2 psychological distress, T2 resilience, and T2 mental well-being was significant (β = −0.459, bootstrapping CI = [−0.572, −0.338]), demonstrating that it was a serial longitudinal mediation relationship.

**Table 2:** Indirect effect of longitudinal serial mediation model

Path	Coeff	95% CI	
		LL	UL
Difficulties in emotion regulation T1 → Psychological distress T2 → Mental well-being T2	-.325	-.440	-.210
Difficulties in emotion regulation T1 → Resilience T2 → Mental well-being T2	-.394	-.525	-.258
Difficulties in emotion regulation T1 → Psychological distress T2 → Resilience T2 → Mental well-being T2	-.459	-.572	-.338

Coeff: Coefficient, CI: Confidence intervals, LL: Lower limit, UL: Upper limit

## Discussion

Difficult life experiences, such as psychological distress, can have a negative influence on the health of people who are not resilient. Individuals with resilience may experience less psychological distress as they adapt to difficult circumstances. Furthermore, people's inability to regulate their emotions may lead to more negative outcomes. This is because each person's subjective experiences are important to their mental health. Natural disasters, such as earthquakes, are among the most severe stressors that can disrupt individuals' psychological well-being. Research has shown that individuals who experience difficulties in emotion regulation are more vulnerable to post-disaster psychological distress, which in turn negatively affects their mental health [28, 41]. Resilience plays a crucial role in mitigating these adverse effects by helping individuals adapt to traumatic events and maintain their psychological stability [42]. The current study used a longitudinal design to investigate the factors influencing people's mental health. Thus, the mediating roles of resilience and psychological distress in the relationship between difficulties in emotion regulation and mental well-being were investigated. The findings of the longitudinal serial mediation analyses revealed that resilience and psychological distress play serial mediation roles between the concepts of difficulties in emotion regulation and mental health. In line with earlier research, those who are more resilient could be better equipped to adjust to natural catastrophes like earthquakes, which would lessen the psychological toll that emotional dysregulation takes [43]. This highlights the significance of resilience-building initiatives, especially for populations that are regularly hit by natural catastrophes. Accordingly, the study's findings were discussed.

The first hypothesis examined is that psychological distress has a mediating role in the relationship between difficulties in emotion regulation and mental well-being (*H1*). Previous research has supported this finding. One of them asserts a positive relationship between difficulties in emotion regulation and psychological distress [44]. In other words, people who struggle to regulate their emotions experience more psychological distress. Researchers have also discovered a mutually negative longitudinal relationship between emotional distress and emotion regulation [45]. On the other hand, both mental well-being and distress have a significant influence on individuals' overall mental health [46]. A study discovered that people with high levels of resilience experienced less psychological distress and reported higher levels of well-being [47]. There are also studies that examine related variables from various perspectives. One of these studies found that difficulties in emotion regulation mediated the relationship between resilience and distress [48]. Gökdağ [49] found that interpersonal emotion regulation predicts psychological distress through social support. Based on these findings, the importance of emotion regulation for individuals seeking improved well-being and reduced psychological distress should not be overlooked. Individuals who do not struggle with emotion regulation can live a mentally healthy, distress-free life. Furthermore, individuals with strong emotion regulation skills may often experience fewer psychological disruptions and achieve a more stable sense of well-being.

The second hypothesis looked at another study finding. Accordingly, resilience serves as a long-term link between difficulty controlling emotions and mental health (*H2*). Emotion regulation predicts resilience and is positively correlated [16, 50, 51]. A study found that responding to stressful situations through emotion regulation is associated with greater resilience [52]. The same study also found a link between emotion regulation strategies and well-being. Research suggests a positive correlation between resilience and well-being [53, 54]. According to Gao et al. [55], resilience, mental health, and well-being are all interconnected, with well-being serving as a mediator between the two. Another study found that emotion regulation predicts life satisfaction both directly and indirectly via resilience [15]. When we consider that the concept of mental well-being refers to the subjective experience of happiness, life satisfaction, and positive psychological functioning, we can conclude that the results of this study support the findings of the current study [8]. Based on all of this, it is clear that for individuals to experience high levels of well-being, they must be able to regulate their emotions easily and thus be more resilient. Hence, the ability to effectively regulate emotions may facilitate greater psychological stability and resilience, facilitating increased overall well-being.

The final and main hypothesis of the current study is that psychological distress and resilience mediate the relationship between difficulties in emotion regulation and mental well-being in a longitudinal study (*H3*). In other words, this finding implies that people will experience less distress, be more resilient, and have better mental health because they can easily regulate their emotions. Examining the valuable studies in the literature revealed that none of them contained all the related concepts together. However, there are studies that discuss some of these concepts together, albeit from different perspectives, and provide support for the current study's findings. One of these studies found that resilience and emotion regulation are important in reducing depression, anxiety, and stress within the context of psychological distress [21]. Similarly, resilience is associated with lower levels of depression and anxiety [56]. Resilience is positively related to distress tolerance while negatively related to difficulties in emotion regulation and distress [57, 58]. While these studies shed light on the links between emotion regulation, resilience, and psychological distress, the relationships between the concepts are similar to those found in the current study. However, research revealed that emotional regulation issues mediated the relationship between psychological distress and resilience [59]. Based on the findings of this study and the previous studies, it is clear that increased psychological distress and decreased resilience, as well as difficulties in emotion regulation, have a negative impact on people's mental health. As a result, it has been observed that people who do not struggle to regulate their emotions experience less distress, are more resilient, adapt to difficulties more easily, and have a high level of mental well-being. This observation underscores the important role played by other variables in improving the mental well-being of individuals who experience disasters such as earthquakes.

## Implications

The current study demonstrated the importance of emotion regulation in avoiding psychological distress, being resilient, and being mentally healthy. Individuals' inability to regulate their emotions can make them more vulnerable in the face of adversity and have a negative influence on their mental health. The significance and necessity of developing emotion regulation skills can be discussed. Group-based acceptance and commitment therapy has been shown to improve difficulties in emotion regulation and distress tolerance [60]. Another study found that emotion regulation training increased individuals' resilience [61]. Moreover, mindfulness-based interventions have been demonstrated to substantially alleviate psychological distress, enhance emotion regulation, and improve overall well-being [62, 63]. On the other hand, well-being therapy, a specialized psychotherapeutic strategy for increasing individuals' well-being and resilience, has been shown to reduce vulnerability to depression and anxiety, both of which are considered distressing [64].

Natural disasters, such as earthquakes, can lead to profound emotional distress and significantly disrupt psychological well-being. Prior research highlights the importance of interventions that emphasize emotion regulation and resilience in alleviating the psychological impact of earthquakes [41, 42]. Post-disaster psychological treatments, including trauma-focused cognitive behavioral therapy (TF-CBT), have proven effective in reducing distress and strengthening coping mechanisms among disaster-affected individuals [65]. Additionally, implementing support groups and resilience-oriented psychoeducation programs in disaster-prone areas may equip individuals with essential coping strategies before a disaster occurs [66]. These findings are consistent with the present study’s results, underscoring the critical role of emotion regulation and resilience in safeguarding mental well-being during and after traumatic experiences.

Aside from these, people who are not feeling mentally healthy can benefit from psychological counseling services. Individuals can learn emotion regulation skills and become more resilient during the psychological counseling process. Counseling is likely to result in high levels of distress and mental well-being. As can be seen, the ability to improve emotion regulation skills and resilience, reduce distress, and increase well-being allows for hope if appropriate interventions are implemented. Finally, the current study's longitudinal design allowed for a deeper understanding of the causal relationship between the variables. Thus, the research findings have greater validity than a cross-sectional design in terms of revealing the relationships between variables.

## Limitations and Future Research

In addition to the significant findings of the current study, some limitations should be noted. First and foremost, the data for the study were collected and analyzed at two distinct time points, three months apart. In future research, data may be collected every five to six months and repeated in multiple waves (e.g., three or four times) to clarify the causal relationship between the relevant variables more precisely. Another limitation of the study is that the data were gathered using self-report scales. Future research may employ a variety of data collection methods. Furthermore, because the data were collected from an adult Turkish sample, their applicability to all cultures and age groups is limited. Furthermore, since the data for this study were gathered in the aftermath of an earthquake, participants' responses may have been influenced by this context. Therefore, future research should investigate similar relationships in different environments and with diverse samples to enhance the generalizability of the findings. As a result, it may be useful in future studies to collect data from various cultures and compare related concepts across cultures, as well as to examine them for individuals of different ages, such as adolescents. Another limitation is the possibility that participants made the social desirability error, despite the fact that the data were collected from volunteers. Therefore, different data collection methods should be preferred in future studies to avoid this error.

## Conclusion

The study's findings revealed that resilience and psychological distress play an important longitudinal serial mediation role in the relationship between difficulties in emotion regulation and well-being. It is critical for people to be able to control their emotions and be resilient. These findings suggest that interventions targeting emotion regulation could be instrumental in reducing psychological distress and fostering resilience. Individuals who improve their emotion regulation skills may experience less psychological distress, become more resilient, and have better mental health. Thus, strengthening emotion regulation abilities may serve as a key strategy for enhancing overall well-being.

## Data Availability

Data will be available on request.

## Declarations

### Pre-Registration Statement

This study was not pre-registered.
